# *MPL* exon 10 mutations in Irish patients with a suspected myeloproliferative neoplasm

**DOI:** 10.17179/excli2021-3454

**Published:** 2021-02-01

**Authors:** Lisa Lee Tokar, Laura Kearney, Stephen E. Langabeer

**Affiliations:** 1 Cancer Molecular Diagnostics, St. James's Hospital, Dublin, Ireland

## ⁯

***Dear Editor,***

The myeloproliferative neoplasms (MPN) of essential thrombocythemia (ET) and primary myelofibrosis (PMF) can be broadly molecularly characterized into four main groups according to the presence of acquired mutations in *JAK2*, *CALR* or *MPL* genes (all resulting in cytokine-independent proliferative signaling) or those without these mutations, termed “triple negative” (Grabek et al., 2020[5]). Of these groups, *MPL* mutations are the less common, occurring in approximately 5-10 % of MPN patients and which result in a particular phenotype (Alvarez-Larran et al., 2018[2]). The majority of *MPL* mutations occur in exon 10 that encodes the transmembrane domain although sporadic mutations elsewhere in the gene have been documented. Within exon 10, the mutational hotspot is the codon for Tryptophan at position W515 (Ma et al., 2011[6]). A review was performed on the frequency and type of *MPL* exon 10 mutations in an Irish population of patients with suspected MPN in order to inform future screening strategies.

**Figure 1:**
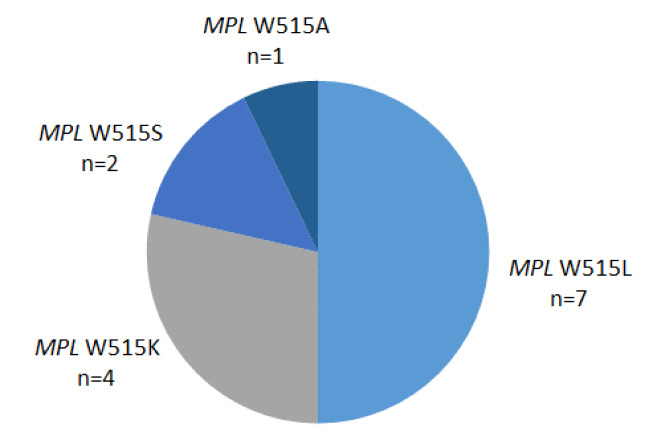
Distribution of *MPL* W515 mutations in an Irish population of patients with a suspected myeloproliferative neoplasm

An audit was performed on all requests for *MPL* exon 10 mutations at a referral center for molecular diagnosis of hematological malignancies over a three-year period from January 2018 to December 2020 inclusive. From this period, 383 requests were received in patients previously characterized as *JAK2* V617F and *CALR* exon 9 mutation undetected. *MPL* exon 10 mutations were detected by a targeted next-generation sequencing (NGS) methodology unchanged throughout the audit period. A total of 14 (3.7 %) of patients had evidence of a W515 mutation comprising *MPL* W515L, W515K, W515S and W515A mutations (Figure 1(Fig. 1)). Absence of clinical details provided means ascribing mutations to either an ET or PMF phenotype is not possible.

These findings are largely in keeping with those from other European populations in respect to both frequency and spectrum of *MPL* exon 10 mutations with the W515L being the most prevalent (Boyd et al., 2010[3]; Cheng Pettersson et al., 2017[4]; Schnittger et al., 2009[7]). An improvement in mutation identification strategy would be to expand NGS coverage enabling routine screening for non-canonical mutations elsewhere in the coding region of *MPL* (Acha et al., 2019[1]). This brief survey provides a platform for continual refinement of the molecular diagnostic algorithm for MPN patients.

## Conflict of interest

The authors declare that they have no conflicts of interest.
